# 59. A rare infective cause presenting as systemic vasculitis a diagnostic dilemma

**DOI:** 10.1093/rap/rky034.022

**Published:** 2018-09-20

**Authors:** Alex Khakwani, Mohammed Abid Yusuf, Magda Al-Zaza, Paul Byrne

**Affiliations:** Rheumatology, Colchester Hospital, Colchester, UNITED KINGDOM

**Introduction:** Rat bite fever (RBF) is a zoonosis and rare presentation to hospital. Its known causative organisms are *Streptobacillus moniliformis* and *Spirillum minus*, the latter of which is almost exclusively restricted to Asia. These organisms are the normal oral and nasopharyngeal flora found within rodents. The most common route of transmission is via bites or scratches, but it has also been noted that it may infrequently be transferred simply by handling rodents. There are approximately 1-2 cases per year reported within the UK. Due to its rarity and remitting non-specific symptoms of pyrexia, headache, myalgia, migratory polyarthralgia and maculopapular rash, it often poses a diagnostic conundrum. It has been noted that the condition can be self-limiting but can also have serious complications such as endocarditis and meningitis. Rapid treatment is therefore of paramount importance, as it has been reported that mortality may reach as high as 7-13% without adequate intervention with antibiotics, such as penicillins, in severe cases. More frequently, this infection may last several months without improvement and may inappropriately be treated as an autoimmune condition. Immunosuppression could worsen prognosis.

**Case description:** A 67 year old white British man presented to the acute medical unit with a one-week history of malaise and difficulty mobilising. He was an ex-smoker and had a background of hypertension and ischaemic heart disease. He had experienced vomiting and diarrhoea, with no blood or mucus in the stool. He had developed bilateral ankle pain over the past few days with swelling, and a bilateral pinpoint purpuric maculopapular rash over the lower limbs developing the day prior to admission. He was not on any regular medication and had no known drug allergies. On admission he had a temperature of 39^o^C but remained haemodynamically stable. It was noted on examination that he had an ejection systolic murmur louder on expiration, but no other abnormalities were found on cardiovascular or respiratory assessment. His abdomen was soft and there was no organomegaly. His right third metacarpophalangeal joint was noted to be swollen and erythematous, as was the left sternoclavicular joint. Both ankles were oedematous and painful. Initial blood samples showed a raised CRP of 271, white cell count (WCC) 15.1, with no other abnormalities. ECG showed sinus rhythm, but the urine alysis was mildly positive for protein and showed trace blood. Due to the development of a previously unknown murmur and pyrexia, he was initially treated for possible infective endocarditis. The rash and urinalysis also led to a differential of autoimmune vasculitis. Initial investigations included three sets of blood cultures and a transthoracic echocardiogram. He was started on intravenous fluid, analgesia and was treated empirically for infective endocarditis according to trust antimicrobial guidelines (vancomycin and gentamicin). A referral was made by the acute physicians to rheumatology at this point. The initial report of the transthoracic ECHO suggested that there may be a small vegetation on the aortic valve with mild mitral regurgitation. Cardiology was consulted and a transoesophageal ECHO was organised, which ultimately showed that there was no vegetation. The initial results of the first blood culture were suggestive of a gram negative rod, so meropenem was started to cover for this organism. Microbiology reviewed the patient and suggested the additional testing of hepatitis A, B, C and HIV, all of which were negative. Rheumatologists reviewed him after this and suggested that an infective cause for the vasculitis was more likely than an autoimmune cause considering the clinical picture and recent blood culture results. Additional tests to include ANCA, ENA, double stranded DNA antibody, anti-CCP antibody and urine protein:creatinine ratio were requested to add to the overall workup, and immunosuppression was not commenced. An ultrasound scan of the left sternoclavicular joint confirmed an inflammatory arthropathy, and a CT thorax showed no obvious abnormalities. *Streptobacillus moniliformis* was isolated from the first blood culture. The patient then declared his hobby of keeping rats and admitted to a recent bite, thus a diagnosis of rat bite fever was made. As he had made a good clinical recovery he was converted to oral amoxicillin and discharged.

**Discussion:** Rat bite fever can be mistaken for vasculitis in view of shared clinical and biochemical features such as a purpuric rash, inflammatory arthritis, cardiac manifestations such as myocarditis, raised inflammatory markers, along with constitutional symptoms including fever, malaise and weight loss. The resultant immunosuppression could worsen prognosis. There is a need to always keep the differential diagnosis as broad as possible when a patient presents with an unusual range of symptoms and signs. Reaching for the most obvious diagnosis before properly working up a patient could lead to a negative outcome. This case illustrates the importance of good clinical history taking and taking a systematic approach to managing any patient. A reasonably robust attempt was made to find a source of infection before instituting immunosuppression.

**Key Learning Points:** A good clinical history is vital for diagnosis and therefore the correct treatment. It should be made more common practice that an animal or other environmental exposure history be included in patients who present with an unusual constellation of symptoms. Rat Bite Fever and other infective arthropathies may mimic the symptomology of vasculitis and other systemic autoimmune conditions, and therefore should be considered as a differential. Early treatment with antibiotics is the most effective way of improving prognosis in these patients.

**Disclosure: A. Khakwani:** None. **M. Yusuf:** None. **M. Al-Zaza:** None. **P. Byrne:** None.

